# Translational Applications of Artificial Intelligence and Machine Learning for Diagnostic Pathology in Lymphoid Neoplasms: A Comprehensive and Evolutive Analysis

**DOI:** 10.3390/biom11060793

**Published:** 2021-05-25

**Authors:** Julia Moran-Sanchez, Antonio Santisteban-Espejo, Miguel Angel Martin-Piedra, Jose Perez-Requena, Marcial Garcia-Rojo

**Affiliations:** 1 Division of Hematology and Hemotherapy, Puerta del Mar Hospital, 11009 Cadiz, Spain; juliamorsan@gmail.com; 2 Ph.D Program of Clinical Medicine and Surgery, University of Cadiz, 11009 Cadiz, Spain; 3 Pathology Department, Puerta del Mar Hospital, 11009 Cadiz, Spain; jose.perez.sspa@juntadeandalucia.es (J.P.-R.); marcial.garcia.sspa@juntadeandalucia.es (M.G.-R.); 4 Institute of Research and Innovation in Biomedical Sciences of the Province of Cadiz (INiBICA), University of Cadiz, 11009 Cadiz, Spain; 5 Tissue Engineering Group, Department of Histology, University of Granada, 18016 Granada, Spain; mmartin@ugr.es

**Keywords:** artificial intelligence, hematopathology, lymphoid neoplasms, digital image analysis, machine learning

## Abstract

Genomic analysis and digitalization of medical records have led to a big data scenario within hematopathology. Artificial intelligence and machine learning tools are increasingly used to integrate clinical, histopathological, and genomic data in lymphoid neoplasms. In this study, we identified global trends, cognitive, and social framework of this field from 1990 to 2020. Metadata were obtained from the Clarivate Analytics Web of Science database in January 2021. A total of 525 documents were assessed by document type, research areas, source titles, organizations, and countries. SciMAT and VOSviewer package were used to perform scientific mapping analysis. Geographical distribution showed the USA and People’s Republic of China as the most productive countries, reporting up to 190 (36.19%) of all documents. A third-degree polynomic equation predicts that future global production in this area will be three-fold the current number, near 2031. Thematically, current research is focused on the integration of digital image analysis and genomic sequencing in Non-Hodgkin lymphomas, prediction of chemotherapy response and validation of new prognostic models. These findings can serve pathology departments to depict future clinical and research avenues, but also, public institutions and administrations to promote synergies and optimize funding allocation.

## 1. Introduction

The storage of clinical information in the electronic medical record and the incorporation of omics data (genomic, transcriptomic, and proteomic) into the patient’s history have led to a novel scenario within pathology departments. Currently, large volumes of information are available for investigators and clinicians, who need to process, integrate, and translate them into daily medical practice.

This data-driven paradigm of 4P medicine (predictive, personalized, preventive, and participative) [1] requires the implementation of computer systems able to process this huge amount of clinical information. In this setting, artificial intelligence (AI) and machine learning (ML) tools have the potential to meliorate diagnostic precision and improve prediction accuracy, and, thus, contribute to a better planification of personalized therapeutic strategies [2].

In recent years, several countries have made public their national research, development, and innovation (RDI) strategies in AI [3,4,5]. The European Union (EU) [6], the United States of America (USA) [7,8,9], and the United Kingdom (UK) [10,11] have communicated their plans for economic coordination in AI and, specifically, an investment increase of 20 billion euros per year is foreseen until 2027 in the EU to develop its coordinated strategy in AI [6].

Among medical disciplines, the field of hematopathology has been pioneering in the application of novel methodologies into the clinical setting, leading to milestones in translational cancer research, such as molecular targeted therapies in chronic myeloid leukemia and acute promyelocytic leukemia, which have rendered curable diseases once considered fatal [12,13].

Regarding this, several works have reported the crescent use of AI and ML tools in the diagnosis of hematological diseases [14,15]. Among hematological malignancies, lymphoid neoplasms (LN) constitute one of the most active foci of research in this area, and different AI algorithms have been developed to improve accuracy in lymphoma subtyping [16,17], validation of prognostic models [18], and prediction of chemotherapy response [19,20]. However, a global analysis of the major trends, leading producers, and scientific mapping of AI and ML applications to diagnostic pathology in LN has not yet been undertaken.

In this study, we evaluated AI and ML applications in LN through bibliometric techniques. Documental evolution, prediction on future production, and leading research centers and countries were identified. Furthermore, we performed scientific mapping analysis (SMA) by means of the packages SciMAT (Science Mapping Analysis Software Tool) [21] and VOS (Visualizing Of Science) viewer [22] to longitudinally assess the cognitive framework and social structure of this research field.

## 2. Materials and Methods

### 2.1. Sample

The metadata used in the study were obtained from the Clarivate Analytics Web of Science (WoS) core collection database of the Thomson Reuters Institute for Scientific Information (ISI) (Philadelphia, PA, USA). Documents were retrieved by searching ((“artificial intelligence” or “deep learning” or “machine learning” or “neural network” or “support vector” or “natural language”) and (“lymphoid neoplasm” or lymphoma or lymphoproliferative or lymphocytic or gammopathy or myeloma or histiocytic)) as topics in the SCI-Expanded Collection for a period range from 1990 to 2020. The dataset was extracted in January 2021 and downloaded in a tab delimited TXT format.

WoS provides information for more than 250 disciplines and, when performing bibliometric analysis, citation data availability is considered one of its main advantages in comparison with other scientific databases such as MedLine [23,24]. Additionally, because of its wide use among the biomedical community, and to evaluate the consistence of our query, we also tested it in PubMed, filtering by article type (journal articles and reviews), subject (cancer; neither AIDS nor complementary medicine), journal (MEDLINE; neither dental journals nor nursing journals), and for the same period (1990–2020). A total of 528 documents was obtained, and we could confirm homogeneity in the retrieved results.

### 2.2. Performance Analysis

Obtained results were classified by document type and then, analysis by research areas, source titles, organizations, and countries was performed on original articles, reviews, proceeding papers, and meeting abstracts, by excluding other minor types such as letters, book chapters, and editorial material.

### 2.3. Science Mapping Analysis (SMA)

SciMAT (Version 1.1.04., University of Granada, Granada, Spain, License GPLv3) and VOS Viewer (Version 1.6.16., Centre for Science and Technology Studies, Leiden University, Leiden, The Netherlands) software were used to perform SMA. In order to achieve a better comprehension of the development of the research field, the analysis was performed for the subperiods: (1) 1990–2005, (2) 2006–2014, and (3) 2015–2020, allowing for a homogeneous distribution of documents.

#### 2.3.1. SMA for Cognitive Framework

SciMAT was employed to longitudinally evaluate the cognitive framework of the area, as previously reported [24]. Briefly, SciMAT uses the author’s keywords (AK) of each article to construct a co-occurrence matrix (CM). Each significant concept (or theme) is defined as the group of AK employed by different researchers during the period analyzed. At this point, the simple center algorithm is applied to construct a thematic network from the CM previously defined. Consequently, the volume of the spheres shown in the strategic diagram is proportional to the number of published articles that contain this specific notion.

Regarding the sources used to construct these strategic diagrams with SciMAT, the package employs the files containing the bibliometric information required, that is, the set of keywords included in each document within the dataset, as it is retrieved from WoS after performing the query term above mentioned. In this sense, a co-occurrence of two keywords during the process of analysis is defined as the joint appearance of two keywords in the same document, as originally conceived by Callon et al. in the seminal papers which laid the foundations of co-word analysis [25,26]. In this study, we employed the simple center algorithm to perform this task, but other approaches were also described such as the single-linkage or sum-linkage [27,28]. Nevertheless, the simple center algorithm has also been validated and employed to identify, characterize, and visualize the cognitive evolution of scientific research in other disciplines [23].

Once these concepts (themes) are obtained, a two-dimensional strategic diagram is depicted based on Callon´s centrality and Callon´s density [25]. These parameters allow a research field to be comprehended as a set of research themes, and SciMAT enabled us to map them into four groups:

(a) Motor themes (MT): Present a high density and a strong centrality signifying the most developed themes for the research area studied (upper-right quadrant);
(b) Basic and transversal themes (BT): Represent themes shared for several disciplines; thus, their foundations are well-established (lower-right quadrant);
(c) Emerging or declining themes (ED): Have a weak density and a low centrality and, thus, represent marginal areas of knowledge (lower-left quadrant);
(d) Highly developed or isolated themes (HDI): Show a high density, meaning a significant internal development. However, they are less connected with other themes in the research field because of their low centrality values (upper-left quadrant).

#### 2.3.2. SMA for Social Framework

VOSviewer was used as previously described by Van Eck and Waltman [22]. VOSviewer allows users to create scientific maps based on network data and exploring them. Its workflow consists of three steps.

First, VOSviewer defines a CM based on the times that any pair of items appear together within the documental corpus. Second, the software applies an algorithm to display a distance-map where each point represents an item in a space. Herein, the VOSviewer uses the SMACOF algorithm to approximate actual Euclidean distances to an ideal model [29]. Third, the maps obtained need to be translated, rotated, and reflected to achieve consistent results.

In our analysis, institutions and countries were evaluated according to its bibliometric coupling relation, that is, the existence of a common cited reference in their reference list [30]. The number of documents published and the number of cites received for each institution or country were employed as weights. Consequently, the final result was a map where the distance between institutions and countries was proportional to its bibliometric coupling relation, and the size of each label in the map was proportional to the number of documents reported or citations received in the period evaluated.

## 3. Results

### 3.1. Performance Analysis

#### 3.1.1. Document Type

**Figure 1:**
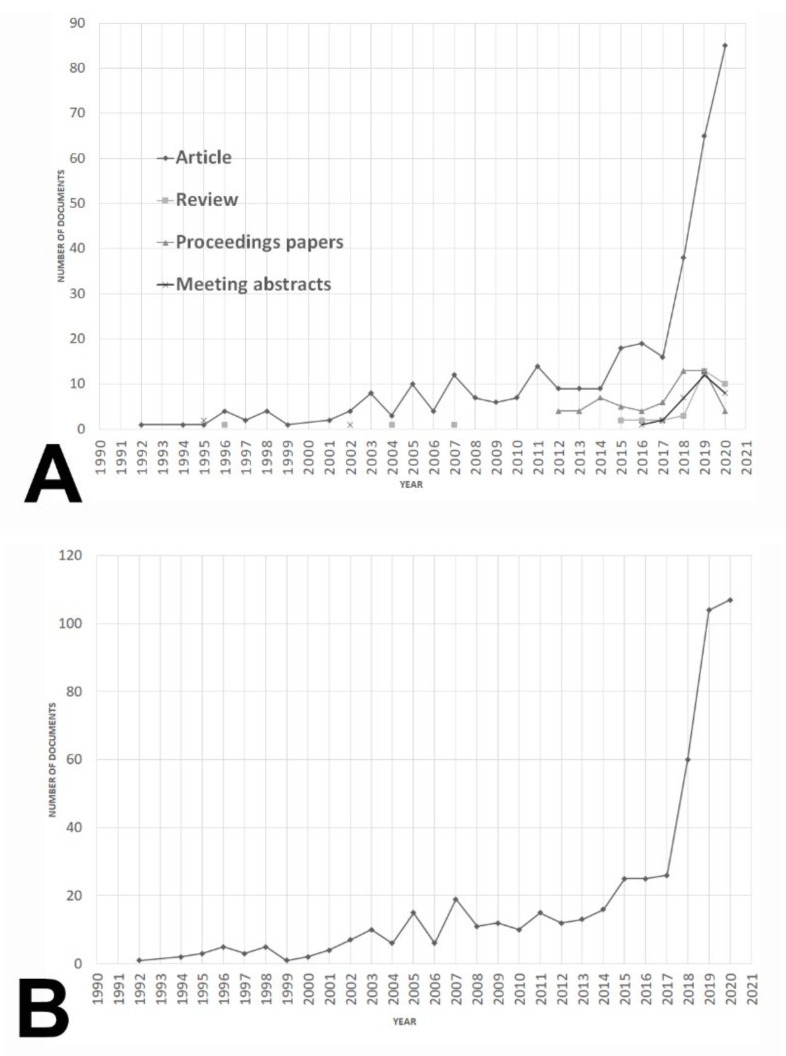
Evolution of documents referred to AI and ML applications to diagnostic pathology in lymphoid neoplasms from 1990 to 2020. (**A**) Document production trends referred to AI and ML applications to diagnostic pathology in lymphoid neoplasms according to document type (original article, review, proceeding papers, and meeting abstracts) from 1990 to 2020. (**B**) Global production trends of documents referred to AI and ML applications to the field of diagnostic pathology in lymphoid neoplasms from 1990 to 2020.

**Figure 2:**
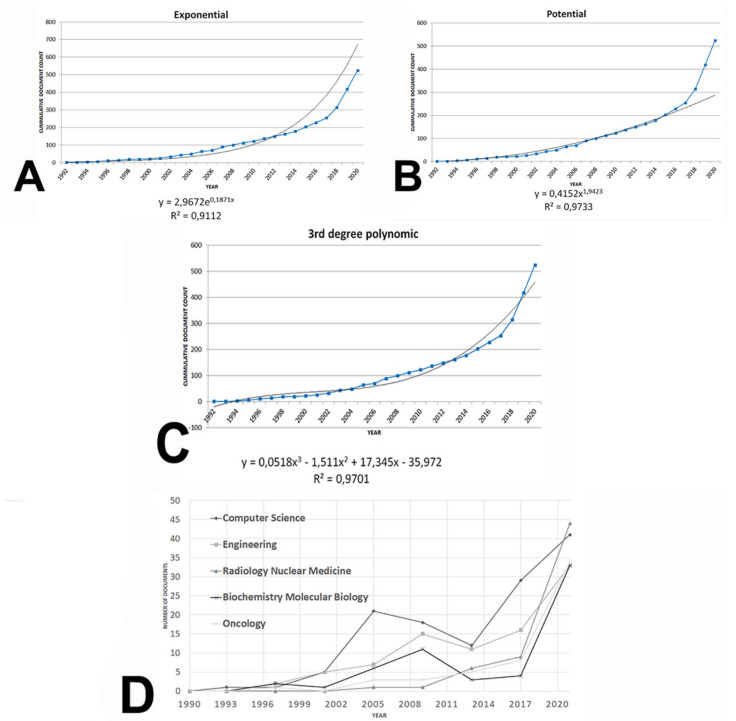
Cumulative journal production by year in the area of AI and ML applications to diagnostic pathology in lymphoid neoplasms. (**A**) Adjustment to an exponential model. (**B**) Adjustment to a potential model. (**C**) Adjustment to a third degree polynomic model. (**D**) Evolution of the five most developed research areas in terms of article production (computer science, engineering, radiology nuclear medicine, biochemistry molecular biology, and oncology) from 1990 to 2020.

A total of 525 documents were retrieved after performing the search strategy. The journal article was the predominant type as it appeared up to 359 documents (68.38% of the corpus), followed by 106 proceeding papers (20.19%), reviews (6.66%), and meeting abstracts (6.28%) (Figure 1A). Furthermore, the growth of the field was particularly remarkable from 2017 to present day (Figure 1B). Cumulative production can be adjusted to an exponential and potential model with a R^2^ = 0.9112 and 0.9733, respectively (Figure 2A,B). Furthermore, a third-degree polynomic model defined by the equation *y = 0.0518x^3^ − 1.511x^2^ + 17.345x − 35.972* (R^2^ = 0.9701) predicted that literature would double in 2027, and it would be three-fold the current number near 2031 (Figure 2C).

#### 3.1.2. Research Areas

During the period analyzed, the research area that gathered most of documents was computer science (CS) with up to 128 documents (24.38%), followed by engineering (EN) with 89 documents (16.95%), radiology nuclear medicine (RNM) with 61 documents (11.61%), biochemistry and molecular biology (BM) with 60 documents (11.43%), and oncology (ON) with 54 documents (10.29%).

Figure 2D shows the evolution of the top five research areas. From 2002 to 2020, CS was at the forefront of research, while ON and RNM increased their contribution to the area since 2008 to the present days. A general crescent trend was evident from 2014 to today both for biomedicine research areas (ON, RNM, and BM) and bioinformatics areas (CS and EN).

#### 3.1.3. Organizations and Research Centers

Table 1 shows the classification of universities and research centers in terms of publications, divided for three subperiods: (1) 1990–2004, (2) 2005–2014, (3) 2015–2020, and globally. Of the 207 documents signed by the 20 most productive institutions, 119 were published in the last period. The University of Texas System (3.62%), the Institute National de la Santé et de la Recherche (INSERM) (2.86%), and Harvard University (2.48%) led global production. Thirteen of the twenty most productive institutions were located in the USA.

The Assistance Publique Hopitaux Paris (APHP) (1.71%), the Centre National de la Recherche Scientifique (CNRS) (1.71%), the Technical University of Munich (1.52%), and the Goethe University of Frankfurt (1.33%) were highlighted within European centers. Finally, the Asiatic contribution showed a crescent trend from the beginning of the study, the Chinese Academy of Science (2.29%) being among the five most relevant centers worldwide for the whole period evaluated.

#### 3.1.4. Source Titles

A total of 397 scientific journals reported at least one of the 525 documents in the area. However, up to 388 journals (96.47%) have published less than five documents, signifying secondary sources. This pattern of scientific production was in accordance with Bradford’s law of bibliographic scattering, which determines that the most of documents belong to a reduced nuclei of core journals, being unproductive to extend literature search beyond it [31].

Table 2 shows the classification of source titles for the period studied. Globally, the most productive journals were Lectures Notes in Computer Science with up to 14 documents (2.67%), Blood with 12 documents (2.28%) and the European Journal of Nuclear Medicine and Molecular Imaging (EJNMMI) with 10 documents (1.90%).

#### 3.1.5. Country Distribution

For the whole period assessed, the USA was the leading producer, reporting up to 190 (36.19%) of all documents. Furthermore, People’s Republic of China with 72 documents (13.71%), Germany with 44 documents (8.38%), India with 35 documents (6.66%), and France with 31 documents (5.90%) were also highlighted as important countries in terms of scientific production (Table 3). Noteworthy, People’s Republic of China and India increased their contributions from 1.56% to 13.71%, and from 1.56% to 6.67%, respectively, during the whole period analyzed.

Worldwide heterogeneity increased as times became nearer to present day, which could be demonstrated by the fact that the number of nations producing more than five documents was 3 (4.84%) in the period 1990–2005, 7 (11.29%) in the period 2006–2014, and 19 (30.65%) in the period 2015–2020.

### 3.2. SMA

#### 3.2.1. SMA for Cognitive Framework

**Figure 3:**
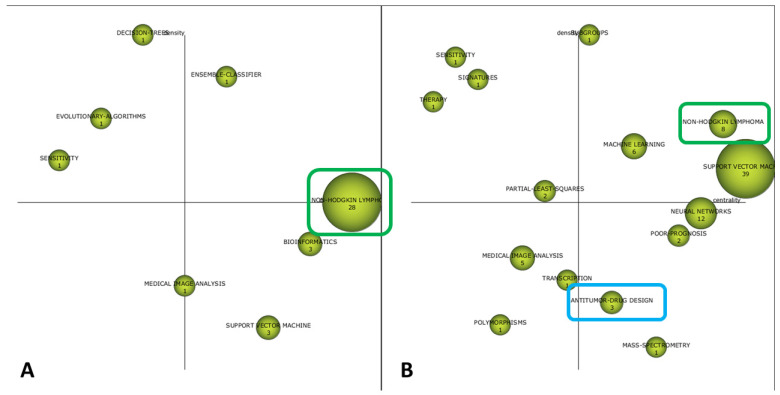
Two-dimensional space layout of research themes on AI and ML applications to diagnostic pathology in lymphoid neoplasms according to Callon´s density (vertical axis) and Callon´s centrality (horizontal axis) as shown by the SciMAT software. Research themes are categorized in Motor themes, Basic and Transversal themes, Emerging or Declining themes, and Highly Developed themes. Some themes that recur over time have been marked in the same color (blue and green). (**A**) Strategic diagram of the cognitive framework for the period 1990 to 2005. (**B**) Strategic diagram of the cognitive framework for the period 2006 to 2014.

**Figure 4:**
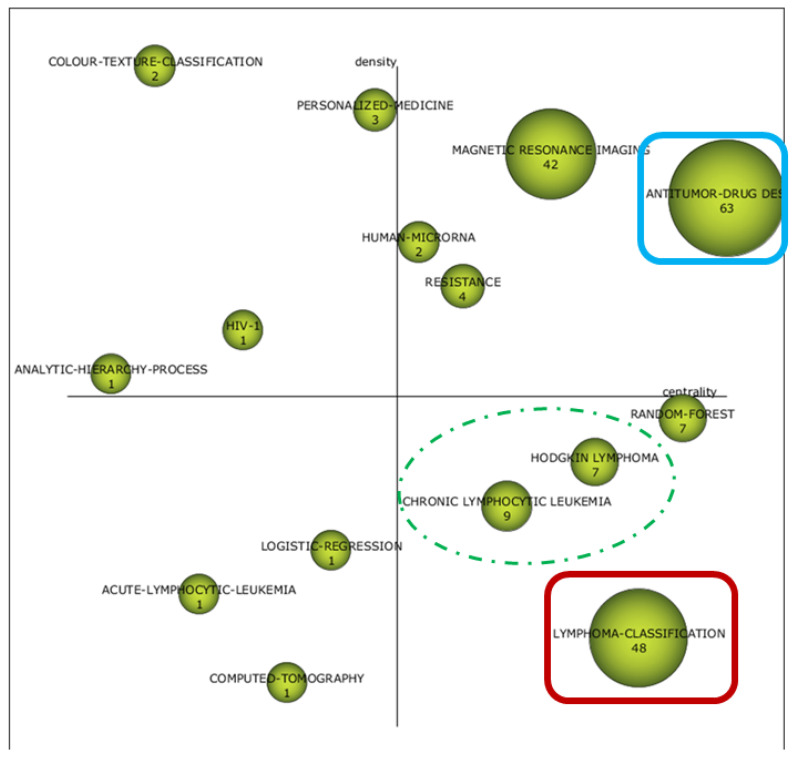
Two-dimensional space layout of research themes on AI and ML applications to diagnostic pathology in lymphoid neoplasms according to Callon´s density (vertical axis) and Callon´s centrality (horizontal axis) as shown by the package SciMAT. Strategic diagram of the cognitive framework for the period 2015–2020. Themes that recur over time have been marked in the same color (blue). The dashed marks in green show clinical entities that have appeared in the last period, compared to non-Hodgkin’s lymphomas in the previous diagrams, also shown in green with continuous lines. The second topic that agglutinates a higher number of documents in the period 2015–2020 (lymphoma classification) has been highlighted in red.

The cognitive framework of the field is shown in Figure 3 and Figure 4. The strategic diagrams show the distribution of themes in MT, BT, HDI, and ED, according to Callon´s density and centrality. Furthermore, the numerical value inside each sphere indicates the number of documents that employ this concept as keyword.

As time approached the present, an increase in the number of terms linked to medical practice took place. First, the ensemble classifier was the only MT identified in the first period, while non-Hodgkin, machine learning, support vector machine, and subgroups were identified as MT in the second period. Moreover, notions such as antitumor drug design, resistance, and magnetic resonance were identified as MT in the last period.

In parallel, BT evolved from the notions of bioinformatics and support vector (period 1990–2005) to neural networks, mass spectrometry, antitumor drug design, and poor prognosis (period 2006–2014). Finally, lymphoma classification, chronic lymphocytic leukemia, Hodgkin lymphoma, and random forest were identified as BT in the period 2015–2020.

#### 3.2.2. SMA for Social Framework

**Figure 5:**
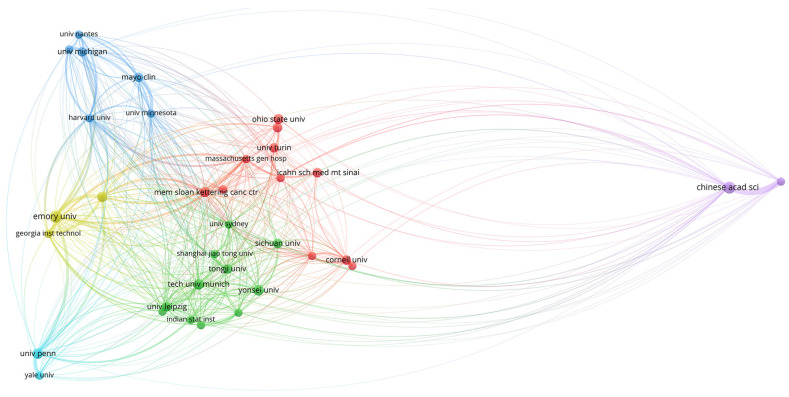
World map showing the structure of relations among research institutions as shown in the network visualization module of the VOSviewer software. The map shows the bibliometric coupling relation among institutions according to the number of documents published for each institution.

**Figure 6:**
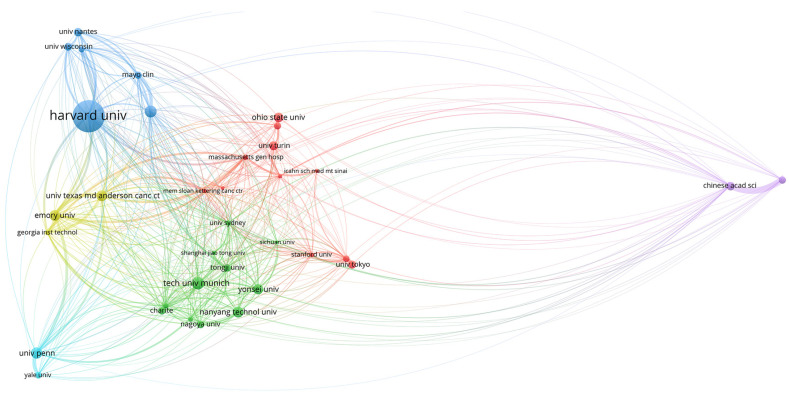
World map showing the structure of relations among research institutions as shown in the network visualization module of the VOSviewer software. The map shows the bibliometric coupling relation among institutions according to the number of citations received for each institution.

**Figure 7:**
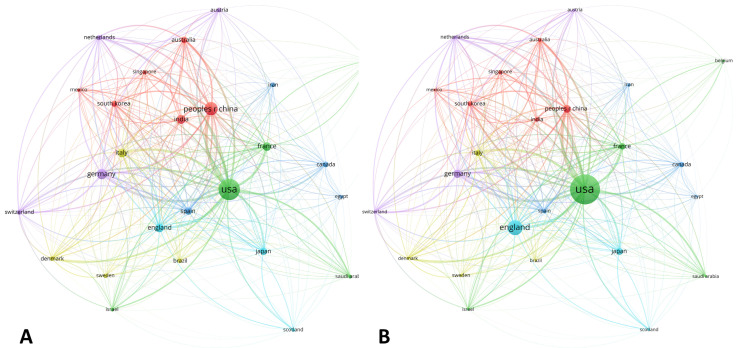
World map showing the structure of relations among countries as shown in the network visualization module of the VOSviewer software. (**A**) Map for the bibliometric coupling relation among countries according to the number of documents published for each country. (**B**) Map for the bibliometric coupling relation among countries according to the number of citations received for each country.

The relations among institutions are shown in Figure 5, Figure 6 and Figure 7. Two different regions can be observed: (1) a cluster of institutions sited in the USA, European centers, and some Asiatic organizations, and (2) an area represented by the Chinese Academy of Science (Figure 5). When citation counts were evaluated, the structure of the map did not change in terms of institutions location; however, the role of Harvard University notably increased (Figure 6).

The global distribution by countries is shown in Figure 7. On the one hand, the USA and People’s Republic of China stood out when documental production was assessed (Figure 7A). Moreover, England, Japan, and a network of European nations (Italy, Spain, Germany, and France) collaborated with the USA, which acted as the central node of the map. On the other hand, the analysis of citation impact revealed some variations, showing a decrease in Asiatic contributions and a maintenance of the USA and England as the major contributors of the area (Figure 7B).

## 4. Discussion

Human reasoning works by integrating new knowledge to previous experience. Thus, it is limited by the volume of information it is able to store and manage. AI, as a computer science aimed to design, develop, and validate devices capable of mimicking human intelligence, has the potential to overcome that barrier. By extension, clinical and diagnostic reasoning could be optimized through the incorporation of AI and ML tools to daily practice in pathology departments.

In the omics era [32], the volumes of data generated by sequencing techniques have led to new insights in the biology of hematological diseases [33], by revolutionizing how clinicians approach diagnosis, prognosis, and make therapeutic decisions. In this sense, research in hematopathology has been pioneering in the translation from bench to bedside [34], allowing for the clinical use of risk stratification models and targeted therapies, based on genetic and molecular data [35,36].

The field of AI and ML applications to diagnostic pathology in LN constitutes an active focus of research [14,15]. Specifically, digitalization of histopathological slides, and the integration of genomic analysis oriented to optimize diagnostic platforms in LN is at the forefront of research [37]. The development of AI/ML methods in other medical disciplines such as oncology is also remarkable. In this sense, in colorectal cancer, some AI algorithms have been developed to automatically discriminate between neoplastic regions and non-tumorous tissue [38]. Using scanned preparations, tumor areas of pancreatic neuroendocrine tumors can be delineated from the stroma using DL [38], which allows better quantification of Ki67 in tumor areas only (97.8% sensitivity, 88.8% specificity) [39]. In breast cancer, it is possible to automatically pinpoint areas of intraductal carcinoma or infiltrating carcinoma on hematoxylin-eosin and classify the digital preparations as benign or malignant, reaching an area under the curve (AUC) of 0.962 in digital preparations [38,39,40].

Of note, the first EU-approved DL system with CE-IVD marking (the official marking required for the European Community) was for the detection of prostate cancer [35]. In the literature, the efficacy in prostate cancer detection of DL systems is remarkably high (using tissue arrays or TMA, prostate biopsies, and prostatectomies), with AUC values of between 0.98 and 0.997 to classify prostate biopsies in benign or malignant [39,41].

However, the identification of the main global trends of AI applications to diagnostic pathology in LN, prediction of future research avenues and definition of its cognitive and social framework had not yet been performed. In this work, we analyzed the status of AI and ML applications to diagnostic pathology in LN by means of bibliometric techniques for a period between 1990 and 2020.

On the one hand, documental production experienced a marked increase, especially since 2017. Based on the behavior of the research field studied, future production can be predicted through a third-degree polynomic equation (*y* = 0.0518*x*^3^ − 1.511*x*^2^ + 17.345*x* − 35.972) which established that literature would double in 2027, and would be three-fold the current number near 2031. This prediction manifestly overwhelms Price´s law of the growth of science, which postulates that publications double each 10–15 years, approximately [42]. Biomedical literature usually does not fit in this model, because of its high consumption and obsolescence rates. Moreover, the use of this equation for monitoring future production should be interpreted cautiously. First, the mere fact of modeling the evolution of science constitutes a challenging activity, as revolutionary ideas usually disrupt the accumulative view of scientific evolution, leading to the emergence of new paradigms that change the way in which research activity is usually conducted, as demonstrated by Kuhn [43]. Furthermore, the mathematical model has certain limitations. In this sense, polynomial regression is a form of linear regression, and consequently the accuracy of predictions, that is, the power of the model to assign a precise value to the dependent variable (i.e., future research production), depends on the number of independent variables included. Subsequently, as new data will be available, there will be the need to update this model. In this sense, not only polynomic models, but ay regression model, will be affected by the availability of new data and, thus, by time. However, the greater the number of terms in the polynomial equation, the greater the accuracy of the model built to provide valuable information. Regarding this, an interesting paper by Ostertagová revised the strengths and drawbacks of the use of polynomial regression for modeling [44].

However, it can be affirmed that the optimization of subtyping in LN by exploding computational analysis of large amounts of genomic information and its integration within platforms of digital image will prominently increase in coming years. This novel scenario will probably put the pathology departments and related colleagues in an unprecedented setting, where basic concepts about bioinformatics and AI will be demanded to professionals in order to achieve an expertise level, both in clinics and research arenas.

Moreover, obtained results about the scientific production in this research field can also be inserted in a Gompertzian model, to better evaluate the current scientific scenario of AI and ML applications to diagnostic pathology in LN [45]. In this way, three stages of scientific evolution can be defined: (1) an initial phase where seminal papers of the area were published, (2) an intermediate phase where an exponential growth of literature occurs leading to the forefront of research, and (3) a last phase characterized by the storage of knowledge by means of reviews and, thus, the consolidation of the field.

Probably, as it can be deduced from Figure 1B, the area of AI and ML applications to diagnostic pathology in LN remains in a transition stage between the second and third phases. It is worthy to note that the rate of document production has been under an exponential model since 2012 (Figure 2A) and, interestingly, the publication of reviews (an early sign of information synthesis) is increasing since 2015 (Figure 1A). Thus, from a global bibliometric perspective, it can be hypothesized that this research field is not in an emerging phase, but rather, its consolidation constitutes an ongoing process.

Globally, most of the documents belong to the area of CS. Nevertheless, RNM has become a major focus of research in recent years. Of note, the integration of molecular imaging and AI algorithms will, probably, constitute an essential pillar in LN management in the near future, not only with diagnostic and staging purposes, but also as a prognostic marker. Regarding this, several AI algorithms have been tested for this purpose, such as deep learning (DL) for the reconstruction of positron emission tomography (PET) image in Hodgkin lymphoma (HL) [46], convolutional neural networks (CNNs) for the prediction of diffuse large B-cell lymphoma (DLBCL) total metabolic tumor based on PET/computed tomography (CT) [47] and support vector machine (SVM) to discriminate hypermetabolic lymphomatous lesions and noncancerous processes [48].

On the other hand, the analysis of source titles shows that three journals, Lecture Notes in Computer Science, Blood, and the EJNMMI, were the most productive journals in the area. In this sense, as there is not a unique bibliographic database covering the whole scientific production in any area, continuous monitoring and updating of the content included in the different available databases are of paramount importance to precisely characterize the scenario where scientific research takes place. In this study, we have used WoS as it constitutes the standard database in bibliometric studies for identifying and monitoring research trends [49]. WoS provides information for more than 250 disciplines, 21,000 scientific journals, and 1.6 billion of cited references from 1900 to the present [24]. However, the list of source titles showed in this work and, hence, the classification of journals could vary if a different bibliographic database is consulted. As a consequence, these results should be interpreted with caution and tacking into consideration the complex process of indexing journal information and bibliometric indicators into the different available bibliographic databases.

Furthermore, our results are in concordance with Bradford’s law of scattering as a minority of sources are responsible for the most of scientific production [28]; in fact, up to 388 journals (96.47%) published less than five documents during the whole period studied. Similar patterns have been demonstrated in other growing areas in biomedicine such as medical advanced therapies [50]. In our study, 103 from the 128 documents retrieved were published in the last period, proving the important editorial effort made in recent years. Regarding this, relevant journals in the area such as Leukemia, Frontiers in Oncology, and the American Journal of Clinical Pathology appeared in the top 20 of source titles.

In terms of contributions, the USA (36.90%), People’s Republic of China (13.71%), Germany (8.38%), India (6.67%), and France (5.91%) were at the front of the research. Among the top five centers worldwide, three of them are located in the USA (University of Texas System, Harvard University, and University of California System), and the INSERM and the Chinese Academy of Sciences were highlighted among European and Asiatic centers, respectively. In this context, the evolution of the People’s Republic of China is remarkable as it has increased its contributions from 1.56% in the first period to 13.71% in the last period, also suggesting an important increase in financial investment in the research area.

Furthermore, we also performed SMA of the field in order to elucidate its cognitive and social framework [51]. Regarding this, several SMA tools have been developed and validated to analyze collaboration patterns among institutions [24,52] , identify growing scientific areas of interest [53] and optimize funding allocation in scientific research [54]. Here, we have used the software SciMAT and VOSviewer, because of their proven quality to perform both conceptual and social evaluation on biomedicine related disciplines [21,22].

First, the cognitive framework of AI and ML applications to diagnostic pathology in LN showed a crescent clinical application of these tools. Interestingly, the ensemble classifier was the only MT identified in the first period. Briefly, EC methods compensate partial errors by introducing the output of one base model as the input for the next algorithm in the sequence, thus, improving the average prediction power [55]. Interestingly, a previous study used this approach to predict mortality after hematopoietic stem cell transplantation (HSCT) [56].

In recent years, most research has been focused on lymphoma subtyping by integrating different AI algorithms. On the one hand, ML, neural networks (NNs), and SVM appeared as MT and BT in the second and third periods, respectively. On the other hand, lymphoma classification was among the most developed concepts in the last period. Specifically, the integration of digital image analysis and genomic sequencing by means of different AI algorithms in non-Hodgkin lymphoma (NHL) constitutes a major topic of research in this area. In this way, logistic regression and Cox proportional hazards have been employed for building a cell-of-origin (COO) classifier in DLBCL based on targeted RNA sequencing (RNA-seq) data [20]. Gene expression profiling (GEP) of 414 DLBCL patients treated with CHOP/R-CHOP were used as inputs for a SVM model which accurately stratified them in two biologically distinct subgroups [57]. Furthermore, a random forest algorithm was trained and validated to discriminate the most frequent B-cell NHL categories among 510 cases of NHL, based on ligation-dependent RT-PCR and next-generation sequencing (NGS) [16].

Moreover, immunophenotyping, either by flow cytometry [58] or immunohistochemistry [59], has been also employed to train AI models for the diagnosis of B-cell NHL. A CNN algorithm was developed based on digital histopathological slides using Aperio ImageScope (Leica Biosystems, Buffalo Grove, IL) to discriminate between Burkitt lymphoma (BL) and DLBCL [60], and interesting approaches grounded on fuzzy logics have demonstrated high accuracy to subclassify DLBCL based on transcriptional profiling data obtained from “lymphochip” DNA microarrays [61].

Drug discovery and prediction of response were also identified as relevant topics in this area. Regarding this, the notion of sensitivity constituted an HDI during the first and second periods, likely accounting for a pre-clinical application of ML to drug evaluation, while antitumor drug design was the most developed concept in the last period. Interestingly, since the original description of DL in drug discovery [62], there have been considerable efforts to expand these kinds of AI applications within hematopathology research. In this setting, Turki et al. developed a transfer learning algorithm to predict sensitivity to Bortezomib in multiple myeloma [63], and a model based on Bayesian network and NNs, combined with RNA-seq, has identified novel mechanisms of resistance in 150 drugs evaluated in DLBCL [64]. Thus, the advent of powerful ML approaches also has the potential to open new horizons in drug evaluation and pharmacogenomic areas [65].

As mentioned, when analyzing research areas and source titles, functional imaging analysis and AI constitutes a growing area of interest. Regarding this, it could be defined a cognitive evolution from general concepts such as medical image analysis (first and second periods) to more specific tools such as magnetic resonance and CT. Herein, deep CNNs have been employed to discriminate patterns of tumor infiltration in PET/CT in 327 patients with NHL [66], and prediction of response to conventional chemotherapy by integrating AI and molecular techniques also constitutes a growing area of interest in recent years [47,67].

In addition, the cognitive evaluation of the field also leads to the identification of particular hematological disorders in which applications of AI and ML are being carried out, such as chronic lymphocytic leukemia (CLL) and Hodgkin lymphoma (HL). Although NHL accounts for the most of research conducted during the whole period, here we also reported ML approaches to identify CLL patients at high risk of infection [68] and to optimize CLL diagnosis through GEP and artificial NNs (ANNs) [69]. In relation to AI and ML applications to the diagnosis of HL, the complexity to adequately isolate Hodgkin and Reed–Stenberg (HRS) cells within a major non-tumoral microenvironment can be under the relative absence of AI applications for this entity. However, stimulating works have proved the potential of ML algorithms to predict prognosis in HL, both in adults [70] and pediatric patients [71].

To better comprehend the structure of this research field, we also evaluated its social framework by means of the software VOSviewer [22]. First, the role of the USA in the development of the area was highlighted both in terms of scientific contributions and citation impact. As depicted in Figure 5, two major nodes of production were identified. On the one hand, there was a cluster of organizations mainly located in the USA and Europe, which also collaborate with certain Asiatic centers (Shanghai Jiao Tong University, Tongji University, Yonsei University, Sichuan University). On the other hand, the Chinese Academy of Sciences appeared relatively isolated towards the periphery of the map.

Obtained results can be explained in terms of different patterns of collaboration, where European and USA institutions tend to a more collaborative trend, while Asiatic centers conduct a more unified research strategy. However, this hypothesis requires more in-depth studies to evaluate the particular structure of each country in terms of scientific investment and science promotion policies. Additionally, when citation impact was assessed, institutions cited in the USA, such as Harvard University, appeared as relevant centers within the map.

In summary, the results of this study show an important increase in scientific production and predict a more accelerated growth over the next 10 years in the field of AI and ML applications to diagnostic pathology in LN. Moreover, the integration of genomic and molecular data with digital image analysis through different AI algorithms will probably constitute an important pilar in the future practice within pathology departments, both to optimize diagnostic and prognostic procedures. In this sense, a better comprehension of the social and cognitive structure of the area can also serve to public institutions and administrations to optimize funding allocation, identify areas of growing interest, and promote synergies among clinical and research centers, which is an unavoidable condition for the right progress of hematopathology.

## 5. Conclusions

The use of AI and ML tools in diagnostic hematopathology is increasing over time, as demonstrated by the crescent trends reported in the literature. On the one hand, most of the research has been focused on the study of non-Hodgkin´s lymphomas in particular, in the analysis of genomic data for improving lymphoma classification, digitalization of histopathological slides, and medical image analysis. On the other hand, different research centers located mostly in Europe and USA highlighted in the social analysis of the research field. Finally, although the results of this work show a growing trend in research and publications on AI applications in the evaluation of LN, the full clinical implementation of these systems in the future will require the training and development of collaborative programs between pathologists, bioinformaticians and clinicians.

## Figures and Tables

**Table 1:** Analysis of documents referred to AI and ML applications to diagnostic pathology in lymphoid neoplasms by institutions from 1990 to 2020. Data are provided by subperiods (1990–2005; 2006–2014; 2015–2020) and globally. C: document count; %: percentage of documents.

Institution	1990–2005		Institution	2006–2014		Institution	2015–2020		Institution	TOTAL	
	C	%		C	%		C	%		C	%
NAGOYA UNIVERSITY	5	7.81	CORNELL UNIV	4	3.50	CHINESE ACAD SCI	10	2.87	UNIV OF TEXAS SYSTEM	19	3.61
AICHI CANC CTR	4	6.25	INDIAN STAT INST	4	3.50	EMORY UNIV	10	2.87	INSERM	15	2.85
ST JOHNS HOSP	3	4.68	JADAVPUR UNIV	4	3.50	UNIV TEXAS MD ANDERSON	8	2.29	HARVARD UNIV	13	2.47
CENT MED LABS	2	3.12	NANYANG TECHNOL UNIV	3	2.63	MEM SLOAN KETTERING CANC CTR	7	2.01	UNIV CALIFORNIA SYSTEM	13	2.47
FLORIDA INT UNIV	2	3.12	NCI	3	2.63	UNIV PENN	7	2.01	CHINESE ACAD SCI	12	2.28
HARVARD UNIV	2	3.12	NIH	3	2.63	ICAHN SCH MED MT SINAI	6	1.72	CORNELL UNIV	12	2.28
NANYANG TECHNOL UNIV	2	3.12	RUTGERS STATE UNIV	3	2.63	MAYO CLIN	6	1.72	UTMD ANDERSON CANCER CENTER	11	2.09
OHIO STATE UNIV	2	3.12	TONGJI UNIV	3	2.63	TECH UNIV MUNICH	6	1.72	EMORY UNIV	10	1.90
THOMAS JEFFERSON UNIV	2	3.12	UNIV MICHIGAN	3	2.63	CHINA UNIV MIN TECHNOL	5	1.43	MEM SLOAN KATTERING CANC CTR	10	1.90
UNIV BIRMINGHAMN	2	3.12	UNIV OXFORD	3	2.63	COLUMBIA UNIV	5	1.43	UNIV PENNSYLVANIA	10	1.90
UNIV MARYLAND	2	3.12	UNIV TOKYO	3	2.63	GEORGIA INST TECHNOL	5	1.43	APHP PARIS	9	1.71
UNIV ROMA LA SAPIENZA	2	3.12	UNIV TURIN	3	2.63	MASSACHUSETTS GEN HOSP	5	1.43	CENT NAT DE LA RECHER SCIENTIFIQUE	9	1.71
UNIV ROMA TOR VERGATA	2	3.12	UNIV ZAGREB	3	2.63	NEW JERSEY INST TECHNOL	5	1.43	SCHOOL OF MED MOUNT SINAI	9	1.71
UNIV SO CALIF	2	3.12	CHARITE	2	1.75	OHIO STATE UNIV	5	1.43	NIH	9	1.71
UNIV TURIN	2	3.12	DANA FARBER CANC INST	2	1.75	SHANGAI JIAO TONG UNIV	5	1.43	MAYO CLINIC	8	1.52
YONSEI UNIV	2	3.12	FLORIDA INT UNIV	2	1.75	SICHUAN UNIV	5	1.43	STATE UNIV SYSTEM OF FLORIDA	8	1.52
BETHESDA HOSP	1	1.56	GOETHE UNIV FRANKFURT	2	1.75	UNIV LEIPZIG	5	1.43	TECH UNIV OF MUNICH	8	1.52
CEDARS SINAI MED CTR	1	1.56	HARVARD UNIV	2	1.75	UNIV SYDNEY	5	1.43	YONSEI UNIV	8	1.52
CENTROL NACL INVEST ONCOL	1	1.56	HOP LYON SUD	2	1.75	YONSEI UNIV	5	1.43	COLUMBIA UNIV	7	1.33
CHINESE PEOPLES LIBERAT ARMY GEN HOPS	1	1.56	INDIAN INST TECHNOL	2	1.75	CHB HOSP	4	1.14	GOETHE UNIV FRANKFURT	7	1.33

**Table 2:** Analysis of documents referred to AI and ML applications to diagnostic pathology in lymphoid neoplasms by source titles from 1990 to 2020. Data are provided for three subperiods (1990–2005; 2006–2014; 2015–2020) and globally. Bold type indicates Bradford nuclei for the 25% of total production for each period. Source titles are abbreviated. C: document count; %: percentage of documents.

Source Title	1990–2005		Source Title	2006–2014		Source Title	2015–2020		Source Title	TOTAL	
	C	%		C	%		C	%		C	%
**ARTIFICIAL** **INTELLIGENCE IN MEDICINE**	3	4.68	**LECTURE NOTES IN COMPUTER SCIENCE**	5	4.38	**EUROP JOURN NUCL MED MOL IMAG**	10	2.87	**LECTURE NOTES IN COMPUTER SCIENCE**	14	2.66
**HUMAN PATHOLOGY**	3	4.68	**BMC** **BIOINFORMATICS**	4	3.50	**BLOOD**	9	2.25	BLOOD	12	2.28
**LECTURE NOTES IN COMPUTER SCIENCE**	3	4.68	PLOS ONE	3	2.63	**SCIENTIFIC** **REPORTS**	8	2.29	**EUROP JOURN NUCL MED MOL IMAG**	10	1.90
PROCEEDINGS OF ANNUAL ICIEE-EMBS	3	4.68	ANALYTICAL CELLULAR PATHOLOGY	2	1.75	PROCEEDINGS OF THE SPIE	7	2.01	PROCEEDINGS OF THE SPIE	9	1.71
BLOOD	2	3.12	ARTIFICIAL INTELLIGENCE IN MEDICINE	2	1.75	JOURNAL OF NUCLEAR MEDICINE	6	1.72	SCIENTIFIC REPORTS	8	1.52
COMPUTATIONAL BIOLOGY AND CHEMISTRY	2	3.12	BMC GENOMICS	2	1-75	LECTURE NOTES IN COMPUTER SCIENCE	6	1.72	BMC BIOINFORMATICS	7	1.33
CYTOMETRY	2	3.12	COMPUTERS IN BIOLOGY AND MEDICINE	2	1.75	COMPUTER METHODS AND PROGRAMS IN BIOMEDICINE	5	1.43	PLOS ONE	7	1.33
JOURNAL OF BIOSCIENCE AND BIOENGINEERING	2	3.12	HEMATOLOGY	2	1.75	FRONTIERS IN ONCOLOGY	5	1.43	ARTIFICIAL INTELLIGENCE IN MEDICINE	6	1.14
NEUROCOMPUTING	2	3.12	IEEE ENGINEERING MBSCP	2	1.75	IEEE ACCESS	5	1.43	JOURNAL OF NUCLEAR MEDICINE	6	1.14
PROCEEDING OF THE 2005 IEE SCIBCB	2	3.12	LEUKEMIA	2	1.75	LABORATORY INVESTIGATION	5	1.43	COMPUTER METHODS AND PROGRAMS IN BIOMEDICINE	5	0.95
2000 IEEE EMBS ICITABMP	1	1.56	PROCEEDING OF THE SPIE	2	1.75	AMERICAN JOURNAL OF CLINICAL PATHOLOGY	4	1.14	FRONTIERS IN ONCOLOY	5	0.95
2001 IEE NUCLEAR SCIENCE SCR	1	1.56	2006 IEEE IJCNNP	1	0.87	BLOOD ADVANCES	4	1.14	IEEE ACCESS	5	0.95
2004 IEE SCBCP	1	1.56	2008 IEEE WORSHOP ON MLSP	1	0.87	CANCERS	4	1.14	JOURNAL OF BIOMEDICAL INFORMATICS	5	0.95
2005 27^TH^ ANNUAL IC-IEE E-EMBS	1	1.56	2008 INTERNATIONAL STCITAB	1	0.87	IEEE ICBB	4	1.14	LABORATORY INVESTIGATION	5	0.95
2005 IEE CSBCP	1	1.56	2009 ANNUAL IC-IEEE-EMBS	1	0.87	JOURNAL OF BIOMEDICAL INFORMATICS	4	1.14	AMERICAN JOURNAL OF CLINICAL PATHOLOGY	4	0.76
2005 IEE NETWORKING SCP	1	1.56	2009 IEEE CONGRESS ON EC	1	0.87	MEDICAL PHYSICS	4	1.14	BLOOD ADVANCES	4	0.76
7^TH^ WORLD CULTICONFERENCE ON SCI.	1	1.56	2010 7^TH^ IEEE ISBINM	1	0.87	PLOS ONE	4	1.14	CANCERS	4	0.76
AMERICAN JOURNAL OF DERMATOPATHOLOGY	1	1.56	2012 7^TH^ ICCCT	1	0.87	CLINICAL CANCER RESEARCH	3	0.86	COMPUTERS IN BIOLOGY AND MEDICINE	4	0.76
AMERICAN JOURNAL OF HEMATOLOGY	1	1.56	2012 9^TH^ IEEE ISBI	1	0.87	GENOME MEDICINE	3	0.86	IEE ICBB	4	0.76
AMIA 2002 SYMPOSIUM PROCEEDINGS	1	1.56	2013 12^TH^ ICMLA	1	0.87	INTERNATIONAL JOURNAL OF LABORATORY HEMATOLOGY	3	0.86	LEUKEMIA	4	0.76

**Table 3:** Analysis of documents referred to AI and ML applications to diagnostic pathology in lymphoid neoplasms by countries from 1990 to 2020. Data are provided for three subperiods (1990–2005; 2006–2014; 2015–2020) and globally. C: document count; %: percentage of documents.

Country	1990–2005		Country	2006–2014		Country	2015–2020		Country	TOTAL	
	C	%		C	%		C	%		C	%
USA	25	39.06	USA	38	33.33	USA	127	36.49	USA	190	36.19
ENGLAND	6	9.37	PEOPLE’S R CHINA	14	12.28	PEOPLE’S R CHINA	57	16.37	PEOPLE’S R CHINA	72	13.71
GERMANY	6	9.37	INDIA	12	10.52	GERMANY	33	9.48	GERMANY	44	8.38
JAPAN	5	7.81	ENGLAND	11	9.64	FRANCE	23	6.60	INDIA	35	6.66
ITALY	4	6.25	ITALY	8	7.01	INDIA	22	6.32	FRANCE	31	5.90
CANADA	3	4.68	FRANCE	7	6.14	SPAIN	19	5.46	ENGLAND	30	5.71
IRELAND	3	4.68	JAPAN	6	5.26	ITALY	18	5.17	ITALY	30	5.71
SINGAPORE	3	4.68	GERMANY	5	4.38	AUSTRALIA	14	4.02	JAPAN	23	4,38
AUSTRALIA	2	3.12	IRAN	5	4.38	ENGLAND	13	3.73	SPAIN	23	4.38
NETHERLANDS	2	3.12	POLAND	4	3.50	JAPAN	12	3.44	AUSTRALIA	17	3.23
NEW ZEALAND	2	3.12	SINGAPORE	4	3.50	SOUTH KOREA	12	3.44	SOUTH KOREA	16	3.04
SOUTH KOREA	2	3.12	CROATIA	3	2.63	CANADA	10	2.87	CANADA	14	2.66
WALES	2	3.12	SPAIN	3	2.63	SWITZERLAND	10	2.87	NETHERLANDS	12	2.28
BARBADOS	1	1.56	AUSTRIA	2	1.75	NETHERLANDS	9	2.58	SWITZERLAND	12	2,28
CROATIA	1	1.56	BRAZIL	2	1.75	AUSTRIA	8	2.29	AUSTRIA	10	1.90
FRANCE	1	1.56	MEXICO	2	1.75	BRAZIL	8	2.29	BRAZIL	10	1.90
INDIA	1	1.56	SLOVENIA	2	1.75	DENMARK	8	2.29	IRAN	10	1.90
ISRAEL	1	1.56	SOUTH KOREA	2	1.75	SWEDEN	7	2.01	DENMARK	9	1.71
PEOPLES R CHINA	1	1.56	AUSTRALIA	1	9.87	SAUDI ARABIA	6	1.72	SINGAPORE	8	1.52
POLAND	1	1.56	BELGIUM	1	0.87	EGYPT	5	1.43	SWEDEN	8	1.52

## Data Availability

Not applicable.
